# O3.5. TESTING THE DOPAMINE HYPOTHESIS OF PSYCHOSIS USING POSITRON EMISSION TOMOGRAPHIC IMAGING IN FIRST EPISODE BIPOLAR AFFECTIVE DISORDER AND SCHIZOPHRENIA

**DOI:** 10.1093/schbul/sby015.203

**Published:** 2018-04-01

**Authors:** Sameer Jauhar, Matthew Nour, Mattia Veronese, Maria Rogdaki, Ilaria Bonoldi, Matilda Azis, Federico Turkheimer, Philip McGuire, Oliver Howes

**Affiliations:** 1 Institute of Psychiatry, Psychology & Neuroscience, King’s College; 2 King’s College London; 3 Northwestern University

## Abstract

**Background:**

The dopamine hypothesis of psychosis suggests that dopamine abnormalities are present in psychotic illness, irrespective of diagnostic class. Meta-analyses of Positron Emission Tomography (PET) studies of the dopamine system have shown elevated dopamine synthesis capacity in schizophrenia, though there is a dearth of studies examining this in other psychotic disorders.

We therefore sought to answer the question of whether abnormalities of the presynaptic dopamine system are seen in bipolar psychosis, how this compared to schizophrenia, and whether positive psychotic symptoms were associated with dopamine synthesis capacity, irrespective of diagnostic class.

**Methods:**

Cross-sectional, case-control 18F-DOPA Positron Emission Tomography (PET) study in people with first episode bipolar psychosis, schizophrenia and control subjects. Clinical measures included the Positive and Negative Syndrome Scale (PANSS), Young Mania Rating Scale and Global Assessment of Functioning (GAF).

**Results:**

Mean (SD) ages were 23.6 (3.6) years in 22 people with bipolar psychosis (13 male), 26.3 (4.4) years in 16 people with schizophrenia (14 male), and 24.5 (4.5) years in controls (14 male). There was a significant group difference in striatal dopamine synthesis capacity (Kicer) (F2,57=6.80, P=.002), post-hoc tests indicating Kicer was significantly elevated in both the bipolar group (mean [SD], 13.18 [1.08]×10–3 min-1; P=.002) and the schizophrenia group (mean [SD], 12.94 [0.79]×10–3 min-1; P=.04) compared with controls (mean [SD], 12.16 [0.92]×10–3 min-1). Kicer was positively correlated with positive psychotic symptom severity in the combined bipolar and schizophrenia sample currently experiencing psychosis, explaining 27% of the variance in symptom severity (n=32, r=0.52, P=.003).

**Discussion:**

This is the first study to examine the presynaptic dopamine system in bipolar psychosis, finding an elevation compared to controls, equivalent to schizophrenia, from first onset of illness. A relationship was found between dopamine synthesis capacity and positive psychotic symptoms, across diagnostic classes, indicating a transdiagnostic role for dopamine synthesis capacity and positive psychotic symptoms.

